# Diagnostic Accuracy and Cost-Effectiveness of Alternative Methods for Detection of Soil-Transmitted Helminths in a Post-Treatment Setting in Western Kenya

**DOI:** 10.1371/journal.pntd.0002843

**Published:** 2014-05-08

**Authors:** Liya M. Assefa, Thomas Crellen, Stella Kepha, Jimmy H. Kihara, Sammy M. Njenga, Rachel L. Pullan, Simon J. Brooker

**Affiliations:** 1 Faculty of Infectious and Tropical Diseases, London School of Hygiene & Tropical Medicine, London, United Kingdom; 2 College of Health Sciences, Makerere University, Kampala, Uganda; 3 Eastern and Southern Africa Centre of International Parasite Control, Kenya Medical Research Institute (KEMRI), Nairobi, Kenya; Instituto de Investigaciones en Enfrmedades Tropicales, Universidad Nacional de Salta, Argentina

## Abstract

**Objectives:**

This study evaluates the diagnostic accuracy and cost-effectiveness of the Kato-Katz and Mini-FLOTAC methods for detection of soil-transmitted helminths (STH) in a post-treatment setting in western Kenya. A cost analysis also explores the cost implications of collecting samples during school surveys when compared to household surveys.

**Methods:**

Stool samples were collected from children (n = 652) attending 18 schools in Bungoma County and diagnosed by the Kato-Katz and Mini-FLOTAC coprological methods. Sensitivity and additional diagnostic performance measures were analyzed using Bayesian latent class modeling. Financial and economic costs were calculated for all survey and diagnostic activities, and cost per child tested, cost per case detected and cost per STH infection correctly classified were estimated. A sensitivity analysis was conducted to assess the impact of various survey parameters on cost estimates.

**Results:**

Both diagnostic methods exhibited comparable sensitivity for detection of any STH species over single and consecutive day sampling: 52.0% for single day Kato-Katz; 49.1% for single-day Mini-FLOTAC; 76.9% for consecutive day Kato-Katz; and 74.1% for consecutive day Mini-FLOTAC. Diagnostic performance did not differ significantly between methods for the different STH species. Use of Kato-Katz with school-based sampling was the lowest cost scenario for cost per child tested ($10.14) and cost per case correctly classified ($12.84). Cost per case detected was lowest for Kato-Katz used in community-based sampling ($128.24). Sensitivity analysis revealed the cost of case detection for any STH decreased non-linearly as prevalence rates increased and was influenced by the number of samples collected.

**Conclusions:**

The Kato-Katz method was comparable in diagnostic sensitivity to the Mini-FLOTAC method, but afforded greater cost-effectiveness. Future work is required to evaluate the cost-effectiveness of STH surveillance in different settings.

## Author Summary

Accurate methods of diagnosis and optimal strategies to sample the population are essential for the reliable mapping and surveillance of infectious diseases. The current standard for detection of soil-transmitted helminths (STH) entails use of the Kato-Katz diagnostic method. Alternative diagnostic methods, such as flotation techniques like the Mini-FLOTAC, have been developed with hopes of achieving greater sensitivity and ease of use. Here, we evaluate the diagnostic accuracy of the Kato-Katz method and the Mini-FLOTAC method for detecting STH infection. We use Bayesian latent class modeling to calculate the diagnostic accuracy in the absence of a gold-standard method for STH detection. Stool samples were collected from school-age children using school-based and community-based sampling. We present cost estimates for use of the Kato-Katz and Mini-FLOTAC diagnostic methods in combination with both sampling methods, providing cost data for the various survey scenarios. Sensitivity was comparable between the Kato-Katz and Mini-FLOTAC methods for detection of any STH species over a single day (Kato Katz: 52.0%, Mini-FLOTAC: 49.1%) and consecutive days (Kato-Katz: 76.9%, Mini-FLOTAC: 74.1%). Costs were lowest in scenarios using the Kato-Katz method; cost per child tested and cost per case correctly classified for school-based sampling with the Kato-Katz diagnostic were $10.14 and $12.84 respectively. The lowest cost per case detected was $128.24 with community-based sampling and use of Kato-Katz. Further work is required on the cost-effectiveness of diagnostic and sampling methods for STH surveys and surveillance of other neglected tropical diseases (NTDs) in various settings. To this end, we provide the model code used in the diagnostic analysis and a costing template for STH surveys.

## Introduction

The reliable mapping, surveillance and evaluation of infectious diseases relies upon two key factors: (i) accurate methods of diagnosis and (ii) optimal strategies to sample the population. For the soil-transmitted helminths (STH: *Ascaris lumbricoides*,*Trichuris trichiura* and hookworm), the commonly used diagnostic technique is the Kato-Katz method [1]. This technique allows for the quantification of intensity of infection on the basis of fecal egg counts. Whilst this method is used widely due to its simplicity and need for minimal equipment, it has low sensitivity arising mainly from the non-random distribution of eggs in stool and day-to-day variation in egg output [2]–[7]. The sensitivity of the method is improved by duplicate readings of samples and collecting samples over consecutive days [8], but this increases effort and cost [9].

An alternative to the Kato-Katz method is a new flotation and translation-based technique, the FLOTAC method [10], which exhibits greater sensitivity for detecting STH species compared to the Kato-Katz method [11]–[14]. However, FLOTAC requires use of a centrifuge which may be unavailable in field laboratories and also consists of more procedural steps. The recently developed Mini-FLOTAC overcomes this constraint and includes a closed chamber for flotation and mixing, and a separate reading disc [15]. A study in Tanzania and India demonstrated that Mini-FLOTAC was more sensitive for STH diagnosis than either a direct smear or the formol-ether concentration technique, while other work has shown Mini-FLOTAC and Kato-Katz to be comparable for hookworm diagnosis in a very high prevalence setting in Tanzania [16].

The choice of diagnostic method should not only take into account ease of use and test performance but should also consider costs [17]. Previous studies have examined the costs of alternative methods for the diagnosis of clinical malaria [18]–[20], but few studies have been conducted for helminth diagnosis. A cost analysis of FLOTAC and Kato-Katz in Zanzibar showed that the additional time requirements for FLOTAC preparation and the specialist equipment required resulted in higher costs compared to the Kato-Katz method [9]. Costs will also be influenced by the sampling platform for the collection of stool samples. Surveys of STH are typically conducted in schools since school-aged children are the natural targets for control and because of the practical advantages of conducting school surveys [1], [21]. However, alternative platforms to schools have recently been proposed for STH surveys, including household surveys conducted as part of the transmission assessment surveys (TAS) used to assess whether lymphatic filariasis is below a pre-defined prevalence threshold [22]. In order to inform the choice of sampling strategy, there is a need to evaluate the relative cost of school-based surveys compared to community surveys.

In the present study, we evaluate the diagnostic accuracy and cost and cost-effectiveness of the duplicate Kato-Katz and Mini-FLOTAC methods in western Kenya where mass treatment had recently been provided as part of the national school deworming programme. Such a low transmission setting will become increasingly important as control programmes effectively reduce infection levels. We use Bayesian latent class models to estimate test sensitivity and specificity in the absence of a gold standard [23]–[25]. This approach has the advantage that it can adjust for the conditional dependence between tests that are based upon a common biological phenomenon (direct observation of eggs) [26]. Our economic analysis not only evaluates the costs and cost-effectiveness of diagnosis, but also explores the cost implications of collecting samples during school surveys compared to household surveys.

## Methods

### Ethics statement

The collection of stool samples and cost data was embedded in a larger study investigating the impact of deworming on malaria-specific immune responses and risk of clinical malaria (ClinicalTrials.gov: NCT01658774) in 20 schools. Written informed consent from child participants was provided by a parent or guardian on the child's behalf. Ethical approval was obtained from the Kenya Medical Research Institute and National Ethics Review Committee (SSC No. 2242), the London School of Hygiene and Tropical Medicine (LSHTM) Ethics Committee (6210), the Makerere School of Public Health, Institutional Review Board (IRB00005876).

### Study area and population

**Figure 1:**
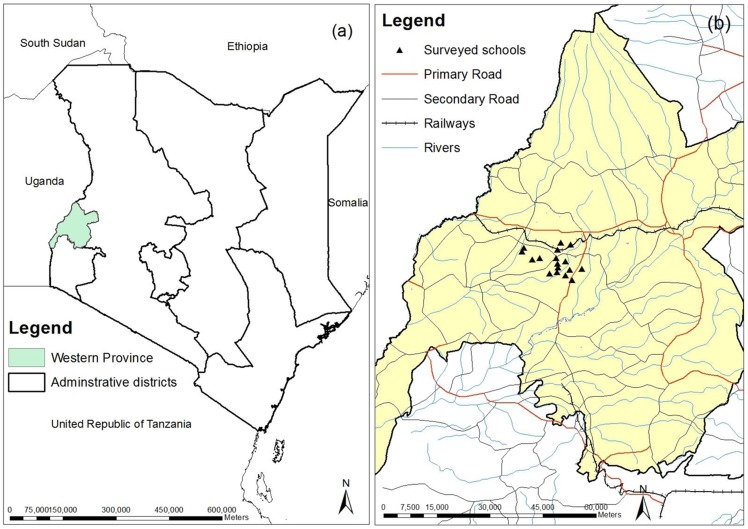
Study site: (a) Western Province within Kenya; (b) Location of the 18 primary schools in Bungoma County, Western Province.

The study took place in Bumula District (0.52747, 34.4395), Bungoma County, Western Province, Kenya (Figure 1) during July 2013. The district is located at 1320 m elevation. Rainfall (annual average of 2428 mm) is seasonally bimodal, with the long rains occurring from March–May and the short rains from October–December. Average annual minimum and maximum temperatures are 11 and 24°C, respectively.

The population of the area consists of indigenous Bukusu people and numerous Luhya who settled in recent years. The economy is primarily rural subsistence agriculture, with some families growing sugar cane as a cash crop. Cattle and sheep are commonly kept. The population is serviced by Bumula District Hospital, which has a catchment area of about 180,000 people and approximately 250 km^2^.

Historically, STH infections were highly prevalent in western and coastal Kenya [27], [28] but a national school deworming programme launched in 2009 has helped to reduce infection levels. As part of this programme, school children were treated with 400 mg of albendazole in June 2013, and thus the study was conducted 20–36 days following delivery of mass treatment delivered through schools. Drug efficacy was not formally investigated as the treatment fell outside of the WHO recommended 14–21 days for assessing anthelmintic drug efficacy [29].

### Data collection

Eighteen schools with the highest prevalence of STH infection, assessed during pre-treatment screening surveys conducted in January 2013, were included in the present study. Data collection was originally planned to take place in schools, however a national teachers strike, June 25–July 17, meant this was not initially possible. So not to delay work, it was decided to collect stool samples from the homes of children enrolled in school for 14 of the 18 schools, with children found to be infected with STH species in the previous screening surveys purposively sampled (household sampled, n = 504). Once the schools reopened, samples from children purposively selected in the remaining four schools were collected at the schools (school sampled, n = 148). These differences in sample collection provided the unforeseen opportunity to estimate the cost of sampling in both households and schools. In a subset of children (n = 233), stool samples were collected over two consecutive days to evaluate if multiple sampling improved test performance.

### Parasitological methods

Each stool sample was examined using the Kato-Katz method and the Mini-FLOTAC method. The Kato-Katz method was performed using a 41.7 mg template, according to the WHO recommendation, and examined in duplicate with different technicians reading each sample. Mini-FLOTAC was performed using 2 g of stool and flotation solution (FS) 2 (saturated sodium chloride) to detect STH [30]. For details on how to conduct each method, see [31]. The intensity of infection was expressed by eggs per gram (EPG) of feces. For the Kato-Katz method, a multiplication factor of 24 was used. For Mini-FLOTAC, a multiplication factor of 10 was used, based on calculation *dilution ratio/volume*, where the dilution ratio is 2 grams of faeces to 38 ml of flotation solution, or 2∶40 (1∶20), and the volume read in the reading disk is 2 ml. 1∶20/2 ml. Quality control was performed on 10% of all samples, where they would be re-examined by a second microscopist to check for discrepancies. Any discrepancies necessitated examination by a third microscopist.

### Analysis of diagnostic accuracy

Results were recorded by hand on data collection sheets and entered into Microsoft Excel version 12 (2007, Microsoft Corporation; Redmond, WA, USA). Statistical analysis was performed on STATA version 10 (College Station, TX, USA) and WinBUGs 1.4.1 software (Imperial College and MRC, UK).

Analysis was based on Bayesian latent class modeling, which is increasingly used to evaluate diagnostic sensitivity for a number of parasite infections, especially in the absence of a ‘gold standard’ reference test [32]–[34]. They are particularly well suited to such problems as they can incorporate prior scientific information about the sensitivities and specificities of the tests and the prevalence of the sampled population, thus overcoming problems of non-identifiability [26], [35]–[37] and can be expanded to account for conditional dependence between tests [35].

In our analysis, each school/community is considered as a separate population *k* with its own (true but unobserved or latent) infection prevalence (π*_k_*). Each population is subjected to two diagnostic tests, *j* (*j* = 1,2); 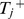
+ and 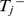 denote positive and negative test results from test *j*, and 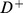 and 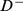 denote true numbers of infected and non-infected. We define *S_j_* and *C_j_* to be the sensitivity and specificity of test *j* where 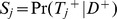 and 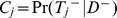; common sensitivities and specificities of each diagnostic test are assumed across all populations, and in the first instance are assumed to be conditionally independent.

Results from each diagnostic test were cross-classified, and the joint distribution assumed to be multinomial with four categories corresponding to all possible combinations of the results in two tests. The multinomial probabilities were expressed as functions of the true prevalence of infection and of the sensitivities and specificities of the two tests. Sensitivity and specificity over two days was considered a direct function of one day sensitivity/specificity. As both tests are based upon a common biological phenomenon (direct observation of eggs) they can be considered conditionally dependent, which must be accounted for in order to obtain unbiased estimates of test accuracy. The models were thus extended to include covariance between tests for infected individuals and for non-infected individuals, following the method of Dendukuri and Joseph [35]. A detailed description of the model is given in Supplementary Information S1.

The diagnostic performance of the methods was further assessed in terms of positive predictive value (PPV, proportion of true positive results detected), negative predictive value (NPV, proportion of true negative results detected) and accuracy (proportion of readings that have given a valid result) based upon modeled prevalence, sensitivity and specificity. Finally, the comparison of methods in estimating EPG was made using the Wilcoxon Rank Sum test.

### Costing data

Financial and economic costs were estimated for diagnosis using both the Kato-Katz and Mini-FLOTAC methods. Since both household and school sampling were undertaken, costs were also estimated for each sampling method.

Costs were estimated using an itemized, ingredients-based approach where individual costs and quantities were recorded [38]. Quantities used for each of the categories were obtained through observation in the field and from accounting records provided by KEMRI. Evaluation of costs was undertaken from the perspective of the provider, here defined as the government. The time frame was for one round of surveillance: nine days for household sampling and four days for school sampling. A wastage value of 10% was applied to all consumables. The costs for all activities were categorized into four categories: personnel, materials, transport and facility. Financial and economic costs were classified separately for each of the cost categories. Financial costs are those that represent the accounting cost of a good or service, representing the actual amount paid. Economic costs can represent opportunity cost, meaning the benefits forgone of a resource not being used in its next best alternative use [39]. Financial and economic costs were combined to provide an overall cost for personnel, materials, transport and facility, for each sampling and diagnostic method.

Costs were collected in Kenyan Shillings and converted to US dollars using an average of the last year of exchange rates, which ranged from $82.23 to $86.80 (www.oanda.com). No annualization or discounting was made due to the time frame of one round of surveillance. All costs obtained were for the year 2013; accordingly no inflation or deflation factor was used in the analysis.

### Cost-effectiveness analysis

Current guidelines for STH control focus on the prevalence levels of any STH species, rather than individual STH species. Therefore, cost-effectiveness was calculated for any STH species rather than for each species. Three outcomes were estimated: (i) cost per sample tested; (ii) cost per case of STH infection detected by each test; and (iii) cost per STH infection correctly classified. Cost per case tested was defined as the total cost of sampling and diagnostic activities per individual tested in the given scenario. Cost per case of STH infection detected was calculated by dividing the total costs for each diagnostic and sampling scenario by the number of positive cases identified in each scenario. Cost per STH infection correctly classified was estimated by dividing the cost per child tested by the related diagnostic test accuracy as estimated in the latent class model.

Probabilistic sensitivity analysis (PSA) allows simulation of a model where uncertain input parameters are sampled within their specified distributions, assessing the combined effect of parameter uncertainty on outcome measures. PSA was conducted to determine how variance in key input parameters affected the cost outcomes of the four survey scenarios. PSA was applied to the cost per case tested, cost per case detected and cost per infection correctly classified. A 10% variance was applied to salaries and per diems of laboratory technicians and per diems of field workers. Cost per case detected will be influenced by the underlying prevalence of infection, therefore prevalence was varied within a distribution of 0–80%. The number of samples collected and diagnosed was varied within the observed maximum and minimum values. Diagnostic test sensitivity and accuracy were also varied within a distribution of observed values in this study and previous studies [16], [30], [40]. Microsoft Excel and Palisade @Risk (www.Palisade.com) were used in the analysis, and @Risk simulations of 1000 iterations were conducted for the PSA of each surveillance scenario.

## Results

A total of 885 stool samples were taken from 652 children from 18 primary schools. Valid comparisons between the Kato-Katz and Mini-FLOTAC methods were made on samples from 525 children: 393 were sampled on a single day and 132 children were sampled on two consecutive days, resulting in 657 samples available for comparison. The primary cause of data loss was insufficient quantity of stool for all three readings (duplicate Kato-Katz, single Mini-FLOTAC) in provided samples. The final survey population had an age range of 5–17 years with a median age of 10 years. Of the 525 children sampled, 286 (54.5%) were male. The prevalence distribution by age and sex is shown in Table 1.

**Table 1:** Prevalence of STH species by sex and age group among school children who were recently dewormed as part of a national deworming programme in Bumula District, western Kenya (n = 525), 2013.

Population characteristics	Number (and %) infected				
	n	Any STH species	Hookworm	*Ascaris lumbricoides*	*Trichuris trichuria*
**Sex**					
Male	286	97 (33.9)	77 (26.9)	14 (4.9)	6 (2.1)
Female	239	64 (26.8)	48 (20.1)	13 (5.4)	3 (1.2)
Total	525	161 (30.6)	125 (23.8)	27 (5.1)	9 (1.7)
**Age (years)**					
5–7	65	26 (30.6)	23 (27.1)	1 (1.2)	2 (2.4)
8–10	188	51 (27.1)	40 (21.3)	8 (4.3)	3 (1.6)
11–13	193	63 (32.6)	47 (24.4)	15 (7.8)	1 (0.5)
14–17	59	21 (35.6)	15 (25.4)	3 (5.1)	3 (5.1)

### Test accuracy

Overall, 93 of the 657 samples were positive for at least one STH species on both tests (14.2%), 485 samples tested negative for any STH species on both tests (73.8%), 45 samples tested positive with Kato Katz and negative with Mini-FLOTAC (6.8%) and 34 tested negative with Kato Katz and positive with Mini-FLOTAC (5.2%). When considering combined results from consecutive days, children were classified as infected if they were positive on either day, and uninfected if testing negative on both days: 23.5% of the 132 children included were classified as positive for any STH species by both tests, 60.6% as negative by both tests, 9.1% as positive by Kato Katz only and 6.8% by Mini-FLOTAC only.

Estimates of sensitivity and specificity for both tests (both singly and over two consecutive days) are provided in Table 2 and further data on PPV, NPV and test accuracy are provided in Supplementary Information. For all three individual STH species, sensitivity and specificity for each test are comparable, lying between 47.3% and 53.3% when conducted just once, and increasing to 72.2–78.2% when conducted on two consecutive days. For diagnosis of infection with any STH species over two consecutive days, the sensitivity estimates for Kato Katz and for Mini-FLOTAC were 76.9% (95% Bayesian credible interval (BCI): 62.2–88.3%) and 74.1% (95% BCI: 59.8–86.6%), respectively. Estimates of specificity were generally above 93%, and even 99% in some instances. Significant correlations between the test outcomes for all three STH species in both infected (*ρ* = 0.37 [95% BCI: 0.06–0.59] for any STH species) and uninfected children (*ρ* = 0.52 [95% BCI: 0.03–0.84]) suggest that the two tests were conditionally dependent, highlighting the inappropriateness of using a combined reference standard for evaluation.

**Table 2:** Sensitivity and specificity of Kato-Katz and Mini-FLOTAC diagnostic methods for soil-transmitted helminths over single and consecutive day sampling as estimated by latent class analysis.

Test	*N* samples/groups	Diagnostic error of Kato Katz (95% BCI)		Diagnostic error of Mini-FLOTAC (95% BCI)	
		Sensitivity	Specificity	Sensitivity	Specificity
**Single day**					
Hookworm	525/18	52.6 (37.8–67.1)	96.5 (92.9–99.3)	47.3 (34.4–60.9)	97.4 (94.5–99.4)
*A. lumbricoides*	525/18	53.3 (33.9–74.2)	99.0 (97.8–99.8)	50.5 (32.2–72.8)	99.4 (98.3–99.9)
*T. trichiura*	525/18	52.9 (37.7–72.5)	99.5 (98.6–99.9)	52.5 (37.3–69.7)	99.6 (98.8–99.9)
Any STH	525/18	52.0 (38.5–65.9)	95.5 (90.8–98.9)	49.1 (36.6–63.4)	96.7 (92.2–99.3)
**Consecutive day**					
Hookworm	132/6	77.6 (61.3–89.2)	93.2 (86.4–98.5)	72.2 (57.0–84.7)	94.9 (89.2–98.8)
*A. lumbricoides*	132/6	78.2 (56.4–93.3)	97.8 (95.6–99.5)	75.5 (54.1–92.6)	98.7 (96.6–99.7)
*T. trichiura*	132/6	77.8 (61.2–92.4)	99.0 (97.3–99.8)	77.4 (60.7–90.9)	99.1 (97.6–99.8)
Any STH	132/6	76.9 (62.2–88.3)	91.2 (82.5–97.8)	74.1 (59.8–86.6)	93.5 (85.2–98.5)

Model results also suggest that PPV, NPV and accuracy do not differ significantly between tests for all three STH species. For *A. lumbricoides* and *T. trichiura*, for which positivity rates were very low (26 and 6 of 657 samples by Kato Katz, 24 and 9 of 657 samples by Mini-FLOTAC, respectively), there was no notable increase in accuracy when the tests were repeated over two consecutive days. However, for hookworm (and thus any STH species), increased sensitivity resulted in some improvement in accuracy when tests were repeated over two consecutive days: for any STH, accuracy increased from 79.0% (95% BCI: 67.1–87.3%) to 85.7% (95% BCI: 76.3–93.3%) for Kato Katz and from 78.8% (95% BCI: 67.5–87.5%) to 85.9% (95% BCI: 76.3–93.3%) for Mini-FLOTAC, although notably the 95% BCI do overlap.

To demonstrate the potentially large influence of prior distributions and assumptions on model parameters and model fit, a sensitivity analysis focusing on the prior distributions of tests' sensitivity and specificity was conducted. Less restrictive prior distributions on specificity resulted in lower values of sensitivity, but did not improve model fit. Choice of prior had less influence on sensitivity and did not qualitatively influence parameter values. Adjusting for conditional dependence for the combined result of two consecutive tests did not improve the model fit, and neither did fitting unique sensitivity/specificity values. Finally, allowing sensitivity and specificity to vary as a function of prevalence did not improve model fit, and resulting parameter estimates remained comparable.

Analysis of EPG estimates demonstrated that there was no statistical difference in the intensity of hookworm infection estimated using the two methods on single sample (*z* = 0.506, *p* = 0.612). Mean EPG increased for each STH species when sampling occurred over two days, but this difference was non-significant (z = −1.78, p = 0.082).

### Cost and cost-effectiveness

Table 3 presents the costs per child tested, cost per case detected (by the given method) and cost per case correctly classified (based on Bayesian latent class modelling) for each diagnostic and sampling scenario, for both single and consecutive day sampling. The Kato-Katz method was cheaper than the Mini-FLOTAC, regardless of the sampling approach (US$ 10.14 vs. US$ 13.11 for school-based sampling and US$11.99 vs. US$14.96 for community-based sampling). Two day sampling doubled the costs per child tested.

**Table 3:** Costs and cost-effectiveness of single and two-day surveys for detection of any STH species infection, based on school-based (SB) or community-based (CB) sampling and use of the Kato-Katz (KK) or Mini-FLOTAC (mF) method, among school children who were recently dewormed as part of a national deworming programme, western Kenya, 2013.

Survey method	Cost per child tested (US$)	No. of positive cases detected^1^ (%)	Cost per positive case detected (US$)^2^	Cost per case correctly classified (US$)^3^
**One day**				
SB + KK	10.14	30 (15.6)	178.66	12.84
SB + mF	13.11	27 (14.1)	234.53	16.64
CB + KK	11.99	71 (18.1)	128.24	15.18
CB + mF	14.96	67 (17.0)	152.04	18.98
**Two day**				
SB + KK	20.28	43 (22.4)	124.65	25.68
SB + mF	26.22	40 (20.8)	158.31	33.27

1 The number of positive cases in each scenario was determined by KK and mF results during one round of community sampling (nine days) and one round of school based sampling (four days).

2 Cost per positive case detected was calculated by dividing the total costs for each diagnostic and sampling scenario by the number of positive cases identified in that scenario.

3 Cost per case correctly classified was estimated by dividing the cost per child tested by the related diagnostic test accuracy as estimated in the latent class model.

Table 3 also presents the number of cases which tested positive for at least one STH, based on each survey diagnostic and sampling scenario and the associated cost per positive case detected. Again, the Kato-Katz method was cheaper than the Mini-FLOTAC method for each sampling method. Interestingly, community-based sampling was associated with lower costs than school-based sampling, due mainly to a greater number of positive cases identified under the community-based sampling.

Latent class analysis of each diagnostic test produced accuracy estimates ranging from 78.8 to 85.9% (Supplementary Information S3). Accuracy for each test was then incorporated in the costing analysis to generate a cost per STH infection correctly classified (either as negative or positive) (Table 3). The cost per case correctly classified was higher than the cost per child tested, but the relative cost-effectiveness of the different diagnostic and sampling scenarios remained comparable, with the Kato-Katz method being the most cost-effective diagnostic method.

### Sensitivity analysis

**Figure 2:**
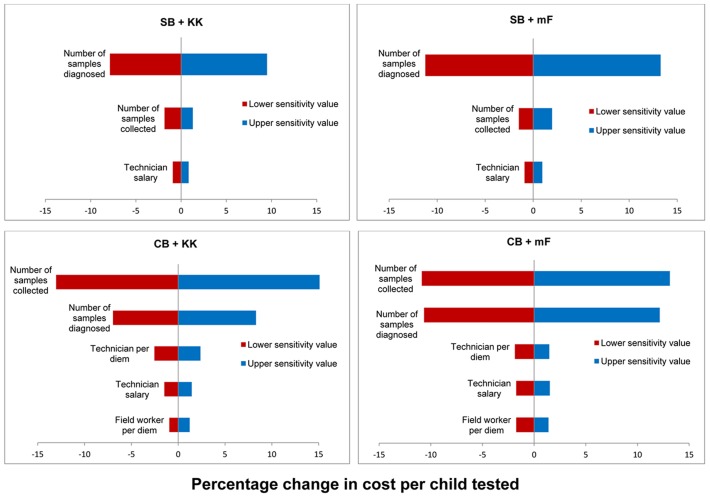
Tornado diagrams of the percentage change in cost per child tested in relation to cost input parameter variation using school-based (SB) and community-based (CB) sampling with diagnosis based on Kato-Katz (KK) and Mini-FLOTAC (mF).

**Figure 3:**
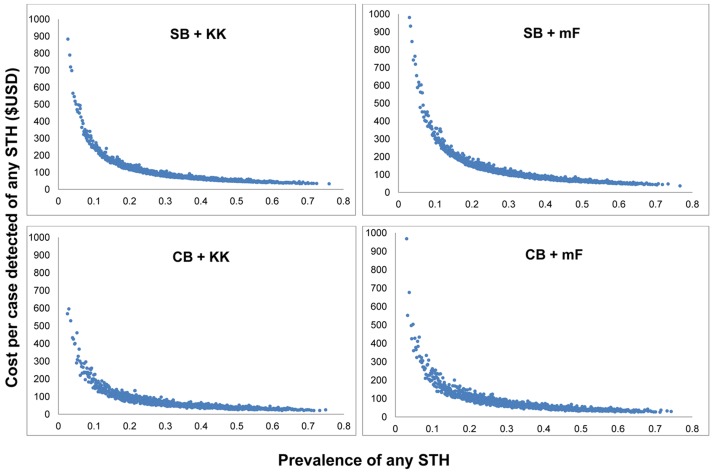
Sensitivity analysis of the relationship between cost per positive case detected and prevalence of infection for alternative diagnostic and sampling methods, using community-based (CB) and school-based sampling (SB) with diagnosis based on Kato-Katz (KK) or Mini-FLOTAC (mF)^1^ among school children who were recently dewormed as part of a national deworming programme, western Kenya, 2013. ^1^ For presentation and comparison purpose, the y-axis for each graph was standardized at $1000. The highest estimate for each scenario was as follows: $1517.48 (SB + KK), $3162.64 (SB + mF), $1929.88 (CB + KK), $1377.23 (CB + mF).

The tornado diagram in Figure 2 presents the percentage change in the cost per child tested and cost per case correctly classified for the input parameters of interest in each survey scenario. Cost per child tested in community sampling was most strongly influenced by the number of household samples collected, while in school-based sampling the cost per child was most strongly influenced by the number of samples diagnosed with either Kato-Katz or Mini-FLOTAC. A similar pattern of parameter influence on cost-effectiveness was observed for the cost per case correctly classified (data not shown). The additional influence of the diagnostic accuracy parameter was noted, although less influential than the aforementioned parameters. Uncertainty in the prevalence estimate for all four scenarios affected most the cost per case of any STH species detected. Sensitivity analysis also demonstrated that the cost per case detected decreased for each scenario as prevalence rates of any STH increased, with costs rapidly declining and eventually reaching a cost threshold (Figure 3).

## Discussion

Suitable surveillance methods are needed to accurately estimate prevalence and intensity of infection, and thus guide disease control programming and track progress towards programme goals. Current recommendations for STH surveys include school-based sampling and parasitological diagnosis using the Kato-Katz method [1], although recently Mini-FLOTAC has been proposed as an alternative technique. Our use of Bayesian latent class modeling showed that in a post-treatment setting in western Kenya, the Kato-Katz and Mini-FLOTAC methods exhibited comparable diagnostic accuracy for detection of any STH species over single and consecutive day sampling. Furthermore, our economic analysis showed that use of the Mini-FLOTAC method was more costly and less cost-effective, whether the samples were collected through school surveys or through household sampling. An advantage of the Mini-FLOTAC method is that it reduces the exposure of the technician to the sample fecal matter, but has the disadvantage of requiring different flotation solutions for STH and for *Schistosoma mansoni*, increasing costs further. In contrast, *S. mansoni* can be read alongside STH using the Kato-Katz method.

The observed sensitivity of each method is lower than that presented in a previous study comparing Kato-Katz and Mini-FLOTAC, which reported a sensitivity of up to 91% and 97% for detecting hookworm using Kato-Katz and Mini-FLOTAC methods respectively [16]. There are two likely reasons for the lower accuracy reported here. First, Barda *et al* conducted their study in a treatment-naïve high intensity setting, whilst infection intensities were considerably lower in the current study. It is widely acknowledged that sensitivity of coprological techniques can be poor in low infection intensity settings. Second, previous analyses have relied on using a combined reference standard. Without a reliable gold standard, the true infection status of a population is unknown, and accordingly, sensitivity and specificity cannot be estimated directly, thus introducing bias in comparing the accuracies of new diagnostic tests. This is especially true when the tests are based on the same biological phenomenon and thus likely to be highly correlated [23], [26]. Bayesian latent class models have the advantage of overcoming this bias by allowing for the estimation of accuracy when true infection prevalence is unknown, under the assumption that sensitivity and specificity are the same in all tested populations. However, intensity of STH and therefore diagnostic sensitivity may differ between populations. It should be noted that simulation studies investigating this approach have shown that when there is a true difference in test sensitivity between populations, results will be biased towards the sensitivity of the test in the population with the highest infection prevalence, thus potentially over-estimating sensitivity in low prevalence settings [41]. Additionally, sensitivity will likely vary across STH species. However, this analysis focused on sensitivity and costs for detection of any STH as that is of primary relevance to STH control and treatment programmes.

The finding that the cost per child tested is lower for Kato-Kato method compared to the Mini-FLOTAC is consistent with a previous costing study in Zanzibar [9]. Our sensitivity analysis illustrated the non-linear trend of decreasing costs with increasing prevalence of infection, with significantly high costs estimated at prevalences below 10%. This result highlights that identifying positive cases will become more expensive as control programmes are successful in reducing infection levels, and programmes and funders should be aware that surveillance costs may increase over the life of a control programme.

Our study additionally provides, for the first time, insight into the cost-effectiveness of diagnosis as previous studies have only estimated costs, and shows that the Kato-Katz method has greater cost-effectiveness in correctly classifying infection status. Previous research on the cost-effectiveness of helminth surveillance has focused on the geographical targeting at the start of control programmes, either in diagnosing individuals infected with *S. haematobium* [42] or sampling strategies for identifying schools requiring mass treatment for *S. mansoni* [43] and STH [44]. The work by Sturrock and colleagues [43], [44] employed Monte Carlo simulation to derive a pseudo gold standard data set, using parameters from empirical data in order capture the spatial and demographic heterogeneities in infection patterns. A similar simulation approach was employed by Smith and colleagues [45] who evaluated the performance of different sampling methods for trachoma surveys. We suggest that the inclusion of diagnostic accuracy, using Bayesian latent class modelling, and the collection of assocated cost data would be an important advance in evaluating the cost-effectiveness of surveillance strategies for STH and other negelcted tropical diseases (NTDs). For example, a combination of simulation, cost-effectiveness and field studies could provide useful insight into the value of transmission assessment surveys for lymphatic filariasis [22] in assessing STH in different epidemiological and programmatic settings.

Notwithstanding the value of our adopted approach, a number of study limitations are worth highlighting. Diagnosis by any microscopy technique is labour intensive and inevitably incurs human error. Although study technicians were rotated to retain alertness, the number of slides processed may still lead to reading errors. This is particularly likely when hookworm is present, as the eggs desiccate after 20–40 minutes in Kato-Katz thick smears [46]. Further limitations are that costs may have been underestimated because personnel performed multiple duties, with technicians undertaking both sample collection and diagnostic preparation. The cost estimates are also limited in their generalizability to an extended time frame of surveillance, as the short survey rounds (four and nine days) cannot provide an estimate of long-term costs. The different sampling methods used (school vs. community-based) suggest differences in cost but not in the parasitological profile of sampled children. The number of positive cases detected in school or community-sampling is unrelated to factors such as school enrolment, as the children sampled by either method were from the same study population, either sampled in their homes or at the schools themselves. Additionally, the distances travelled from school or community sampling locations to the diagnostic facility did not vary enough to alter transport costs. Daily fuel expenditure was equal across both sampling methods, so fuel costs were not significantly influenced by variation in sampling method. Finally, the recent national deworming may have affected the number of positive cases identified by either diagnostic method, influencing costs for case detection. In regard to the generalisability of findings, the relationship between costs and prevalence as shown in Figure 3 suggests that costs of detection would decrease with increasing prevalence of infection, and thus the costs of surveys conducted in high transmission are likely to be lower than the results presented here.

In conclusion, our evaluation shows that the Kato-Katz and Mini-FLOTAC methods were comparable to one another in diagnostic sensitivity, yet Kato-Katz afforded greater cost-effectiveness. We encourage the wider use of simulation, cost-effectiveness and field studies to evaluate the cost-effectiveness of diagnostic and sampling strategies for STH surveillance in a variety of settings and for the wider surveillance of different NTDs. To this end, we provide the code for the Bayesian latent class modeling (Supplementary Information S1) and a costing template for use in future studies (Supplementary Information S2).

## Supporting Information

Supplementary Information S1 STARD diagnostic flowchart. *Of the 525 children included, 393 children were single day sampled and 132 children were consecutive day sampled, providing a total of 657 samples for single day comparison.
(PDF)

Supplementary Information S2 STARD checklist.
(DOC)

Supplementary Information S3 Bayesian latent class model.
(DOCX)

Supplementary Information S4 Additional diagnostic results.
(DOCX)

Supplementary Information S5 Template for the collection of STH survey costs.
(DOCX)
