# Supplementary figures and images for: Diagnostic Accuracy and Cost-Effectiveness of Alternative Methods for Detection of Soil-Transmitted Helminths in a Post-Treatment Setting in Western Kenya
Source: PLoS Negl Trop Dis. 2014 May 8;8(5):e2843. doi: 10.1371/journal.pntd.0002843 (PMC4014443; doi:10.1371/journal.pntd.0002843)

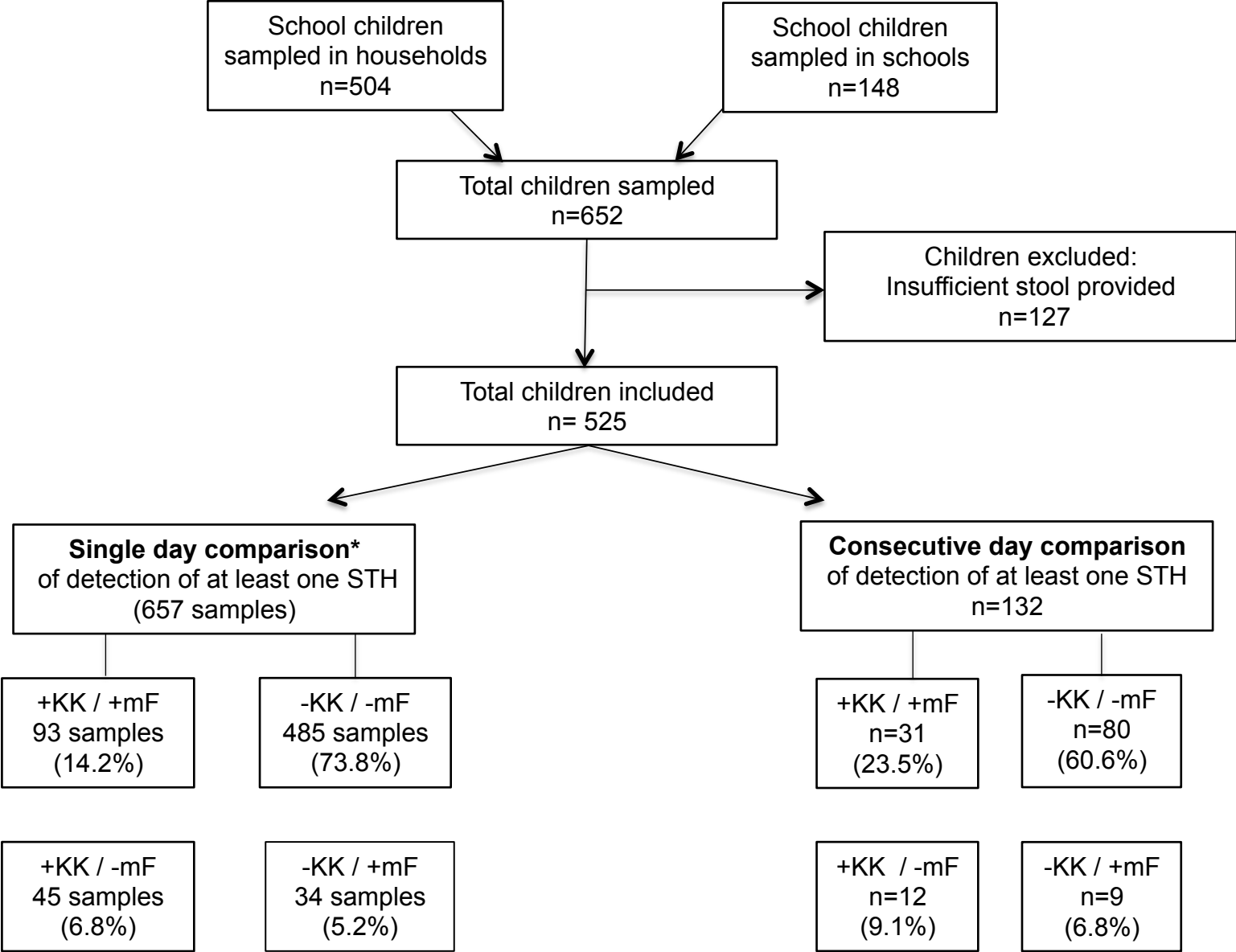

Supplement: Supplementary Information S1 — STARD diagnostic flowchart. *Of the 525 children included, 393 children were single day sampled and 132 children were consecutive day sampled, providing a total of 657 samples for single day comparison. (PDF) [file pntd.0002843.s001.pdf]
