# Supplementary material for: Diagnostic Accuracy and Cost-Effectiveness of Alternative Methods for Detection of Soil-Transmitted Helminths in a Post-Treatment Setting in Western Kenya
Source: PLoS Negl Trop Dis. 2014 May 8;8(5):e2843. doi: 10.1371/journal.pntd.0002843 (PMC4014443; doi:10.1371/journal.pntd.0002843)
Supplement: Supplementary Information S3 — Bayesian latent class model. (DOCX) [file pntd.0002843.s003.docx]

**Supplementary Information S3**

**Bayesian Latent Class Model**

We use latent class models to estimate test accuracy in the absence of a gold standard (1, 2). We adjust for the possibility of conditional dependence between ≥2 tests in ≥2 populations using Bayesian inference, as proposed by Dendukuri and Joseph [[45](#_ENREF_45)], building on previous work [[46](#_ENREF_46)] [[47](#_ENREF_47)] [[48](#_ENREF_48)].

In this analysis, we evaluate the accuracy of two methods for STH, Kato-Katz and Mini-FLOTAC, based on a sample of 525 individuals from 18 schools/communities. For 12 schools/communities, data were available from one day only, whilst for six schools tests were repeated on consecutive days increasing the number results per school to three (day 1, day 2 and combined). Here, we investigate positivity for each of the STH species individually (hookworm, *Ascaris lumbricoides* and *Trichuris trichiura)* and positivity for any STH species. When considering results from consecutive days, individuals were classified as infected if they were positive on either day, and uninfected if testing negative on both days.

For the analysis, we consider each school/community as a separate population *k* with its own (true but unobserved) infection prevalence (π*_k_*). Each population is subjected to two diagnostic tests, *j* (*j* = 1,2); $T_{j}^{+}$and $T_{j}^{-}$denote positive and negative test results from test *j,* and $D^{\mp}$and $D^{-}$denote true numbers of infected and non-infected. We define *S_j_* and *C*$j$ to be the sensitivity and specificity of test *j* where $S_{j}=\Pr(T_{j}^{+}|D^{+})$ and $C_{j}=\Pr(T_{j}^{-}|D^{-})$; common sensitivities and specificities of each diagnostic test are assumed across all populations, and in the first instance are assumed to be conditionally independent. Two-day sensitivity was defined based as 1 – (1-*S_j_*)*(1-*S_j_*) and two-day specificity as *C_j_*C_j._* For each population, results from each diagnostic test were cross-classified in 2x2 tables as follows:

|  |  | ***J_1_*** | |
| --- | --- | --- | --- |
|  |  | *+* | - |
| ***J_2_*** | + | X*_k++_* | X*_k+-_* |
|  | - | X*_k-+_* | X*_k--_* |

The joint distribution of the results ($X_{k++},X_{k\pm},X_{k-+},X_{k--}$) of the two tests is multinomial, ${\left( X_{k++},X_{k\pm},X_{k-+},X_{k--} \right)\sim Multi(p}_{k++},p_{k\pm},p_{k-+},p_{k--}, N_{k})$ with the multinomial probabilities calculated as shown below using the law of total probability:

$$p_{k++}=P\left( T_{1}^{+}, T_{2}^{+}| kth population \right)={[S}_{1}S_{2}]\pi_{k}+[\left( 1-C_{1} \right)\left( 1-C_{2} \right)](1-\pi_{k})$$

$$p_{k+-}=P\left( T_{1}^{+}, T_{2}^{+}| kth population \right)={[S}_{1}\left( S_{2}-1 \right)]\pi_{k}+[\left( 1-C_{1} \right)C_{2}](1-\pi_{k})$$

$$p_{k-+}=P\left( T_{1}^{+}, T_{2}^{+}| kth population \right)=\left[ \left( S_{1}-1 \right)S_{2} \right]\pi_{k}+{[C}_{1}\left( 1-C_{2} \right)](1-\pi_{k})$$

$$p_{k--}=P\left( T_{1}^{+}, T_{2}^{+}| kth population \right)=\left[ \left( S_{1}{-1)(S}_{2}-1 \right) \right]\pi_{k}+{[C}_{1}C_{2}](1-\pi_{k})$$

The model described above assumes that both tests are independent from one another conditional on the true disease status. However, given that they are both based upon a common biological phenomenon this assumption is unacceptable and may lead to misleading results. For example, those individuals experiencing high worm burdens are also likely to have high faecal egg counts, making infection easier to detect by either method. Dendukuri and Joseph (2001) generalized the Bayesian inference approach described above to allow for conditional dependence by directly incorporating the covariance between the two tests (ref). Specifically, the covariance between tests for infected individuals is given by ${cov}_{D^{+}}=S_{12}- S_{1}S_{2}$, where $S_{12}=\Pr(T_{1}^{+}T_{2}^{+}|D^{+})$, and for non-infected individuals by ${cov}_{D^{-}}=C_{12}- C_{1}C_{2}$ where $C_{12}=\Pr(T_{1}^{-},T_{2}^{-}|D^{-})$. By this parameterization, the probability of a concordant result in an infected individual is increased by an amount ${covD}^{+}$, and in a non-infected individual by ${cov}_{D^{-}}$. Conversely, the probabilities of discordant results are penalized by the same amount. Multinomial probabilities are thus revised to include the additional parameters:

$$p_{k++}=P\left( T_{1}^{+}, T_{2}^{+}| kth population \right)={[S}_{1}S_{2}+{covD}^{+}]\pi_{k}+[\left( 1-C_{1} \right)\left( 1-C_{2} \right)+{covD}^{-}](1-\pi_{k})$$

$$p_{k+-}=P\left( T_{1}^{+}, T_{2}^{-}| kth population \right)={[S}_{1}\left( S_{2}-1 \right)-{covD}^{+}]\pi_{k}+[\left( 1-C_{1} \right)C_{2}-{covD}^{-}](1-\pi_{k})$$

$$p_{k-+}=P\left( T_{1}^{-}, T_{2}^{+}| kth population \right)=\left[ \left( S_{1}-1 \right)S_{2}-{covD}^{+} \right]\pi_{k}+{[C}_{1}\left( 1-C_{2} \right)-{covD}^{-}](1-\pi_{k})$$

$$p_{k--}=P\left( T_{1}^{-}, T_{2}^{-}| kth population \right)=\left[ \left( S_{1}{-1)(S}_{2}-1 \right)+ {covD}^{+} \right]\pi_{k}+{[C}_{1}C_{2}+{covD}^{-}](1-\pi_{k})$$

The conditional correlation between the two test outcomes for infected individuals is given as:

$$\rho_{D^{+}}=\frac{covD^{+}}{\sqrt{S_{1}\left( {1-S1}_{1} \right)S_{2}(1-S_{2})}}$$

And for non-infected individuals as:

$$\rho_{D^{-}}=\frac{covD^{-}}{\sqrt{C_{1}\left( {1-C}_{1} \right)C_{2}(1-C_{2})}}$$

Finally, positive predictive value (PPV), negative predictive value (NPV) and accuracy for each test were estimated based upon overall prevalence (π), and test sensitivity and specificity:

$$\pi= \frac{\sum\pi_{k}N_{k}}{\sum N_{k}}$$

$${PPV}_{j}= \frac{S_{j}\pi}{S_{j}\pi+{(1-C}_{j})(1-\pi)}$$

$${NPV}_{j}= \frac{C_{j}\pi}{C_{j}(1-\pi)+{(1-S}_{j})\pi}$$

$${Accuracy}_{j}= S_{j}\pi+C_{j}(1-\pi)$$

Models were fit using MCMC simulation methods in the Winbugs 1.4.1 software (Imperial College and MRC, UK). The two sensitivities and two specificities were assumed to have independent prior distributions and the two covariances were assumed to be distributed according to generalized beta distributions, specified as uniform priors that satisfy the statements: $\left( S_{1}-1 \right)\left( 1-S_{2} \right)\leq{covD}^{+}\leq\min\left( S_{1},S_{2} \right)-S_{1}S_{2}$ and $\left( C_{1}-1 \right)\left( 1-C_{2} \right)\leq{covD}^{-}\leq\min\left( C_{1},C_{2} \right)-C_{1}C_{2}$. Uncertainty around the values of π*_k_*, and the two sensitivities and two specificities were modeled using independent beta prior distributions. Reasonably informative prior information is required for at least four of the seven model parameters because of the non-identifiability of the model. In this instance, specificity of both tests is assumed to be high as experienced technicians are unlikely to mistake the shape of an egg. Thus, the beta distributions used for specificity of both tests were selected to have a mode = 0.95 and 5^th^ percentile 0.7; informative (mode = 0.7 and a 5^th^ percentile at 0.5) and non-informative gamma priors were used for sensitivity, and a non-informative gamma prior for $\pi_{k}$. Following a burn-in of 9,000 iterations, the values for the intercept and coefficients were stored for 1,000 iterations and model convergence was assessed using diagnostic tests and by visually inspecting the time series plots. Convergence was successfully achieved after 10,000 iterations, and the model was run for a further 5,000 with thinning every five, during which predictions were made.

A sensitivity analysis was performed to investigate model assumptions and the influence of prior distributions. Model fit was compared using the DIC.

The code for the final model is given below:

**MODEL {**

for (i in 1:6) { # (for each of the schools with two days)

# results for day 1:

y1[i,1] <- (prev[i]*(Se[1]*Se[2] + CovSe)) + (((1-prev[i])*(1-Sp[1])*(1-Sp[2]) + CovSp)) # positive in both

y1[i,2] <- (prev[i]*(Se[1]*(1-Se[2]) - CovSe)) + (((1-prev[i])*(1-Sp[1])*Sp[2] - CovSp)) # positive in test1

y1[i,3] <- (prev[i]*((1-Se[1])*Se[2] - CovSe)) + (((1-prev[i])*Sp[1] * (1-Sp[2]) - CovSp)) # positive in test2

y1[i,4] <- (prev[i]*((1-Se[1])*(1-Se[2]) + CovSe)) + (((1-prev[i]) *Sp[1]*Sp[2] + CovSp)) # negative in both

result1[i,1:4] ~ dmulti(y1[i,1:4], testedb[i]) # observed data

# results for day 2:

y2[i,1] <- (prev[i]*(Se[1]*Se[2] + CovSe)) + (((1-prev[i])*(1-Sp[1])*(1-Sp[2]) + CovSp)) # positive in both

y2[i,2] <- (prev[i]*(Se[1]*(1-Se[2]) - CovSe)) + (((1-prev[i])*(1-Sp[1])*Sp[2] - CovSp)) # positive in test1

y2[i,3] <- (prev[i]*((1-Se[1])*Se[2] - CovSe)) + (((1-prev[i])*Sp[1] * (1-Sp[2]) - CovSp)) # positive in test2

y2[i,4] <- (prev[i]*((1-Se[1])*(1-Se[2]) + CovSe)) + (((1-prev[i]) *Sp[1]*Sp[2] + CovSp)) # negative in both

result2[i,1:4] ~ dmulti(y2[i,1:4], testedb[i]) # observed data

# aggregated results:

yb[i,1] <- (prev[i]*(Se[3]*Se[4] + CovSe2)) + (((1-prev[i])*(1-Sp[3])*(1-Sp[4]) + CovSp2)) # positive in both

yb[i,2] <- (prev[i]*(Se[3]*(1-Se[4]) - CovSe2)) + (((1-prev[i])*(1-Sp[3])*Sp[4] - CovSp2)) # positive in test1

yb[i,3] <- (prev[i]*((1-Se[3])*Se[4] - CovSe2)) + (((1-prev[i])*Sp[3] * (1-Sp[4]) - CovSp2)) # positive in test2

yb[i,4] <- (prev[i]*((1-Se[3])*(1-Se[4]) + CovSe2)) + (((1-prev[i]) *Sp[3]*Sp[4] + CovSp2)) # negative in both

resultb[i,1:4] ~ dmulti(yb[i,1:4], testedb[i]) # observed data

totalb[i] <- prev[i] * testedb[i] # true infected

}

Se[3] <- (1-((1-Se[1])*(1-Se[1]))) # Se[3] as a function of Se[1]

Sp[3] <- Sp[1]*Sp[1]

Se[4] <- (1-((1-Se[2])*(1-Se[2]))) # Se[4] as a function of Se[2]

Sp[4] <- Sp[2]*Sp[2]

for (i in 1:12) { # (for each of the schools with only 1 day)

y[i,1] <- (prevA[i]*(Se[1]*Se[2] + CovSe)) + (((1-prevA[i])*(1-Sp[1])*(1-Sp[2]) + CovSp)) # positive in both

y[i,2] <- (prevA[i]*(Se[1]*(1-Se[2]) - CovSe)) + (((1-prevA[i])*(1-Sp[1])*Sp[2] - CovSp)) # positive in test1

y[i,3] <- (prevA[i]*((1-Se[1])*Se[2] - CovSe)) + (((1-prevA[i])*Sp[1] * (1-Sp[2]) - CovSp)) # postivie in test2

y[i,4] <- (prevA[i]*((1-Se[1])*(1-Se[2]) + CovSe)) + (((1-prevA[i]) *Sp[1]*Sp[2] + CovSp)) # negative in both

result[i,1:4] ~ dmulti(y[i,1:4], tested[i]) # observed data

total[i] <- prevA[i] * tested[i] # true infected

}

sum.total <- sum(total[1:12]) + sum(totalb[1:6])

sum.tested <- sum(tested[1:12]) + sum(testedb[1:6])

Pi <- sum.total/sum.tested

for (i in 1: 4) {

PPV[i] <- (Se[i] * Pi) / ((Se[i]*Pi) + ((1-Sp[i])*(1-Pi)))

NPV[i] <- (Sp[i] * (1-Pi)) / ((Sp[i] * (1-Pi)) + ((1 -Se[i]) * Pi))

Accuracy[i] <- (Se[i]*Pi) + (Sp[i]*(1-Pi))

}

**## Priors**

for (i in 1:12) { prevA[i] ~ dbeta(1,1) }

for (i in 1:6) { prev[i] ~ dbeta(1,1) }

for (i in 1:2 ) {Se[i] ~ dbeta(13.3,6.3) }

for (i in 1:2 ) {Sp[i] ~ dbeta(21.2,2.06) }

CovSe ~ dunif(SeL, SeU)

CovSp ~ dunif(SpL, SpU)

CovSe2 ~ dunif(SeL2, SeU2)

CovSp2 ~ dunif(SpL2, SpU2)

SeL <- (Se[1]-1)*(1-Se[2])

SeU <- min(Se[1],Se[2]) - (Se[1]*Se[2])

SeL2 <- (Se[3]-1)*(1-Se[4])

SeU2 <- min(Se[3],Se[4]) - (Se[3]*Se[4])

SpL <- (Sp[1]-1)*(1-Sp[2])

SpU <- min(Sp[1],Sp[2]) - (Sp[1]*Sp[2])

SpL2 <- (Sp[3]-1)*(1-Sp[4])

SpU2 <- min(Sp[3],Sp[4]) - (Sp[3]*Sp[4])

rhoDplus <- CovSe / sqrt(Se[1] * (1-Se[1])*Se[2]*(1-Se[2]))

rhoDminus <- CovSp / sqrt(Sp[1] * (1-Sp[1])*Sp[2]*(1-Sp[2]))

**}**
