# Supplementary material for: Diagnostic Accuracy and Cost-Effectiveness of Alternative Methods for Detection of Soil-Transmitted Helminths in a Post-Treatment Setting in Western Kenya
Source: PLoS Negl Trop Dis. 2014 May 8;8(5):e2843. doi: 10.1371/journal.pntd.0002843 (PMC4014443; doi:10.1371/journal.pntd.0002843)
Supplement: Supplementary Information S4 — Additional diagnostic results. (DOCX) [file pntd.0002843.s004.docx]

**Supplementary Information S4**

**Additional diagnostic results**

Table 1. Diagnostic results for Kato-Katz and Mini-FLOTAC for single and consecutive day sampling of soil-transmitted helminths as estimated by latent class analysis

1. **Kato Katz: Single day**

|  | Hookworm | *A. lumbricoides* | *T. trichiura* | Any STH |
| --- | --- | --- | --- | --- |
| Sensitivity | 0.526 (0.378-0.671) | 0.533 (0.339-0.742) | 0.529 (0.377-0.725) | 0.520 (0.385-0.659) |
| Specificity | 0.965 (0.929-0.993) | 0.990 (0.978-0.998) | 0.995 (0.986-0.999) | 0.955 (0.908-0.989) |
| PPV | 0.879 (0.745-0.975) | 0.857 (0.696-0.967) | 0.976 (0.933-0.996) | 0.875 (0.731-0.968) |
| NPV | 0.813 (0.669-0.897) | 0.948 (0.877-0.980) | 0.849 (0.736-0.927) | 0.773 (0.614-0.873) |
| Accuracy | 0.822 (0.709-0.896) | 0.942 (0.880-0.972) | 0.868 (0.769-0.934) | 0.790 (0.671-0.873) |
| Correlation between tests: | |  |  |  |
| Infected children | 0.363 (0.071-0.557) | 0.739 (0.384-0.928) | 0.377 (-0.043-0.583) | 0.370 (0.055-0.592) |
| Uninfected children | 0.284 (-0.013-0.687) | 0.388 (0.021-0.818) | 0.481 (0.039-0.904) | 0.521 (0.032-0.843) |

1. **Mini-FLOTAC: Single day**

|  | Hookworm | *A. lumbricoides* | *T. trichiura* | Any STH |
| --- | --- | --- | --- | --- |
| Sensitivity | 0.473 (0.344-0.609) | 0.505 (0.322-0.728) | 0.525 (0.373-0.697) | 0.491 (0.366-0.634) |
| Specificity | 0.974 (0.945-0.994) | 0.994 (0.983-0.999) | 0.996 (0.988-0.999) | 0.967 (0.922-0.993) |
| PPV | 0.898 (0.762-0.976) | 0.901 (0.742-0.981) | 0.978 (0.939-0.996) | 0.990 (0.755-0.981) |
| NPV | 0.798 (0.659-0.881) | 0.945 (0.877-0.972) | 0.848 (0.731-0.729) | 0.762 (0.603-0.864) |
| Accuracy | 0.813 (0.704-0.775) | 0.942 (0.880-0.975) | 0.867 (0.767-0.933) | 0.788 (0.675-0.874) |
| Correlation between tests: | |  |  |  |
| Infected children | 0.363 (0.071-0.557) | 0.739 (0.384-0.928) | 0.377 (-0.043-0.583) | 0.370 (0.055-0.592) |
| Uninfected children | 0.284 (-0.013-0.687) | 0.388 (0.021-0.818) | 0.481 (0.039-0.904) | 0.521 (0.032-0.843) |

1. **Kato Katz: Consecutive days**

|  | Hookworm | *A. lumbricoides* | *T. trichiura* | Any STH |
| --- | --- | --- | --- | --- |
| Sensitivity | 0.776 (0.613-0.892) | 0.782 (0.564-0.933) | 0.778 (0.612-0.924) | 0.769 (0.622-0.883) |
| Specificity | 0.932 (0.864-0.985) | 0.978 (0.956-0.995) | 0.990 (0.973-0.998) | 0.912 (0.825-0.978) |
| PPV | 0.847 (0.693-0.965) | 0.815 (0.638-0.958) | 0.967 (0.909-0.994) | 0.842 (0.683-0.959) |
| NPV | 0.897 (0.760-0.962) | 0.975 (0.917-0.995) | 0.923 (0.819-0.977) | 0.870 (0.715-0.949) |
| Accuracy | 0.877 (0.781-0.845) | 0.957 (0.909-0.982) | 0.931 (0.854-0.977) | 0.857 (0.763-0.933) |

1. **Mini-FLOTAC: Consecutive days**

|  | Hookworm | *A. lumbricoides* | *T. trichiura* | Any STH |
| --- | --- | --- | --- | --- |
| Sensitivity | 0.722 (0.570-0.847) | 0.755 (0.541-0.926) | 0.774 (0.607-0.909) | 0.741 (0.598-0.866) |
| Specificity | 0.949 (0.892-0.988) | 0.987 (0.966-0.997) | 0.991 (0.976-0.998) | 0.935 (0.852-0.985) |
| PPV | 0.872 (0.714-0.970) | 0.872 (0.697-0.974) | 0.972 (0.917-0.995) | 0.873 (0.709-0.974) |
| NPV | 0.879 (0.745-0.946) | 0.972 (0.915-0.994) | 0.922 (0.813-0.977) | 0.860 (0.707-0.942) |
| Accuracy | 0.873 (0.780-0.934) | 0.961 (0.910-0.986) | 0.931 (0.949-0.974) | 0.859 (0.763-0.933) |
