# Supplementary material for: Diagnostic Accuracy and Cost-Effectiveness of Alternative Methods for Detection of Soil-Transmitted Helminths in a Post-Treatment Setting in Western Kenya
Source: PLoS Negl Trop Dis. 2014 May 8;8(5):e2843. doi: 10.1371/journal.pntd.0002843 (PMC4014443; doi:10.1371/journal.pntd.0002843)
Supplement: Supplementary Information S5 — Template for the collection of STH survey costs. (DOCX) [file pntd.0002843.s005.docx]

**Supplementary Information S5**

**Template for the collection of STH survey costs**

|  | **Item** | **Unit** | **Unit cost (local)** | **Unit cost ($US)** | **Quantity** |
| --- | --- | --- | --- | --- | --- |
|  |  |  |  |  |  |
| **MATERIALS** |  |  |  |  |  |
| **Sample collection** | Stool polypot | item |  |  |  |
|  | Wooden spatulas | box |  |  |  |
|  | Toilet paper | roll |  |  |  |
|  | Newspaper | pack |  |  |  |
|  | Plastic bag | Item |  |  |  |
|  | Disposable gloves | box |  |  |  |
|  | Permanent marker | Item |  |  |  |
|  | Cooler box | Item |  |  |  |
|  |  |  |  |  |  |
| **Diagnostics** | Microscope | Item |  |  |  |
|  | Microscope slides | box |  |  |  |
|  | Microscope counter | Item |  |  |  |
|  | Stool sieves | Item |  |  |  |
|  | Stool templates | Item |  |  |  |
|  | Disposable gloves | box |  |  |  |
|  | Cellophane | Roll |  |  |  |
|  | Malachite green | bottle |  |  |  |
|  | Glycerine | bottle |  |  |  |
|  | Forceps | Item |  |  |  |
|  | Extension cable | Item |  |  |  |
|  | Labels | pack |  |  |  |
|  | Markers | item |  |  |  |
|  | Newspaper | pack |  |  |  |
|  |  |  |  |  |  |
| **Cleaning** | Bleach | bottle |  |  |  |
|  | Liquid detergent | bottle |  |  |  |
|  | Towel | item |  |  |  |
|  | Bucket | item |  |  |  |
|  | Brushes | item |  |  |  |
|  | Gloves | box |  |  |  |
|  | Bin bags | pack |  |  |  |
|  |  |  |  |  |  |
| **Data Collection** | Pencil | item |  |  |  |
|  | Eraser | item |  |  |  |
|  | Sharpener | item |  |  |  |
|  | Pen | item |  |  |  |
|  | Recording sheets | item |  |  |  |
|  |  |  |  |  |  |
| **PERSONNEL** | Head coordinator | per diem or salary | |  |  |
|  | Field worker | per diem or salary | |  |  |
|  | Laboratory technician | per diem or salary | |  |  |
|  | Driver | per diem |  |  |  |
|  | Cleaner | per diem |  |  |  |
|  | Village elder | per diem |  |  |  |
|  |  |  |  |  |  |
| **TRANSPORT** | Vehicle | rental or purchase | |  |  |
|  | Insurance | annual |  |  |  |
|  | Maintenance | per km |  |  |  |
|  | Fuel | litre |  |  |  |
|  |  |  |  |  |  |
|  |  |  |  |  |  |
| **FACILITY** | Office rental | per day or annual value | |  |  |
|  | Laboratory rental | per day or annual value | |  |  |
|  | Building utilities |  |  |  |  |
